# Pirenzepine Reverses Chemotherapy-Induced Peripheral Neuropathy in Mice

**DOI:** 10.3390/pharmaceutics18101233

**Published:** 2026-09-29

**Authors:** Markos Kassahun, Alireza Tayarani, Kyle Freedman, Sebastian Gastelum, Rakesh Nemmani, Jacob Andrew, Meryem H. Soylu, Katie E. Frizzi, Angela Hansen, Paul Fernyhough, Corinne G. Jolivalt, Dwayne G. Stupack, Nigel A. Calcutt

**Affiliations:** 1 Department of Pathology, University of California San Diego, San Diego, CA 92093, USA; 2 Department of Ob/Gyn & Reproductive Sciences, University of California San Diego, San Diego, CA 92093, USA; 3 WinSanTor Inc., 7220 Trade St., San Diego, CA 92121, USA; 4 Division of Neurodegenerative and Neurodevelopmental Disorders, St. Boniface Hospital Albrechtsen Research Centre, R4046—351 Taché Ave, Winnipeg, MB R2H 2A6, Canada; 5 Department of Pharmacology and Therapeutics, University of Manitoba, Winnipeg, MB R3E 0T6, Canada

**Keywords:** chemotherapy-induced peripheral neuropathy, CIPN, muscarinic receptor antagonist, allodynia, peripheral neuropathy, neuropathic pain, pirenzepine

## Abstract

**Background/Objectives:** Mitochondrial dysfunction is a common feature of many peripheral neuropathies and agents that enhance mitochondrial function have potential as pan-neuropathy therapeutics. M1 receptor (M1R) antagonists enhance mitochondrial function and reverse multiple indices of neuropathy in rodents and humans with diabetic neuropathy. The present studies extend evaluation of the M1R-selective antagonist pirenzepine to mouse models of chemotherapy-induced peripheral neuropathy (CIPN), with a primary focus on pain relief. **Results:** Oxaliplatin-exposed mice developed allodynia to light touch that persisted for months before resolution. Systemic pirenzepine (1 mg/kg/day) starting prior to chemotherapy did not prevent allodynia but accelerated recovery. Topical delivery (4% pirenzepine in hydrogel) to the paw, beginning after onset of allodynia, also accelerated recovery, with efficacy persisting weeks after treatment cessation and accompanied by normalization of paw responses to heat. Allodynia in paclitaxel-exposed mice was also reversed, but not prevented, by systemic or topical pirenzepine, while heat hyperalgesia and nerve conduction slowing were dose-dependently normalized. Efficacy of pirenzepine was not associated with disruption of the cytotoxicity of chemotherapeutics, as pirenzepine did not alter the LD50 of paclitaxel when applied to breast or ovarian cancer tumor lines in vitro. Tumor suppression by a cisplatin/paclitaxel cocktail given to mice seeded with OVCAR-8 ovarian cancer cells was also not impacted by pirenzepine. In contrast, pirenzepine did not prevent or reverse allodynia caused by the proteasome inhibitor bortezomib. **Conclusions:** These studies position pirenzepine as a novel interventional treatment against established neuropathy caused by taxanes and platin-based agents and may guide design of therapeutic regimens for future clinical studies.

## 1. Introduction

Chemotherapy-induced peripheral neuropathy (CIPN) is a debilitating side effect of many first-line chemotherapeutic drugs (reviewed in [1]). Depending on the specific drug and dose regimen, CIPN can afflict between 20 and 85% of cancer patients receiving these drugs and approaching 100% of patients when high drug doses are employed. CIPN is frequently first described as a distal sensory neuropathy that presents as numbness and tingling indicative of sensory loss due to distal degenerative peripheral neuropathy. Many patients with CIPN also experience neuropathic pain, presenting as allodynia, hyperalgesia and/or spontaneous pain, while motor and autonomic neuropathy may also be detected upon examination. Signs and symptoms of neuropathy may resolve with reduction or cessation of chemotherapy, but in many instances persist for months to years after treatment has concluded, leading to extended quality-of-life disruption [2]. The onset of CIPN also may precipitate changes to the chemotherapeutic regimen such as reductions in dose, frequency or duration of treatment with the goal of reducing symptoms, particularly pain, to a tolerable level. CIPN therefore both impedes quality of life and compromises effective cancer treatment. The American Society of Clinical Oncology makes no recommendations for the prevention of CIPN and provides only a moderate recommendation for treatment with duloxetine, a serotonin–norepinephrine reuptake inhibitor, for symptomatic relief of pain [3]. Given that more than half a million cancer patients suffer CIPN, this neuropathy remains a major unmet clinical concern. Identifying an adjuvant therapy that prevents or reverses CIPN would enable oncologists to administer more effective chemotherapeutic regimens.

Each class of chemotherapeutic agents has distinct mechanisms by which they impact cancer. For example, the antineoplastic effects of taxanes and vinca alkaloids include disruption of microtubules, while platinum-based drugs create DNA adducts, and bortezomib disrupts proteasomal degradation [1]. While features of CIPN may also reflect drug-specific mechanisms, preclinical studies suggest that multiple chemotherapeutic classes, including taxanes such as paclitaxel, platins such as oxaliplatin, and proteasome inhibitors such as bortezomib, share a common feature of mitochondrial damage and disrupted cell energetics in neurons that ultimately leads to retraction/degeneration of distal regions of the axon [4]. The convergence on mitochondrial dysfunction as a pathogenic mechanism for neurotoxicity also extends to other common peripheral neuropathies, such as those experienced following HIV infection or onset of diabetes [5,6]. Agents that protect neurons from mitochondrial damage or enhance mitochondrial supply and function have the potential to treat many common and currently underserved peripheral neuropathies.

We have previously demonstrated that structurally diverse antagonists of the muscarinic type 1 receptor (M_1_R) enhance mitochondrial activity and axonal growth mechanisms in adult sensory neurons in vitro [7]. Recent mechanistic studies indicate that enhanced mitochondrial activity is downstream of a canonical consequence of M_1_R antagonism that promotes calcium influx and activation of the calcium/calmodulin-dependent protein kinase β (CaAMKK β) and downstream AMP-activated protein kinase (AMPK) pathway [8,9,10]. While mitochondrial activation can provide additional energetic support for axonal growth, the physical process of elongation driven by antagonist binding to the M_1_R is mediated via β-arrestin-dependent biased agonism [11,12]. A therapeutic application arising from delineation of these pathways is suggested by preclinical studies in rodent models of diabetes, which show that systemic or topical delivery of M_1_R antagonists results in the prevention and reversal of multiple functional and structural indices of peripheral neuropathy [7,13,14,15]. Translational relevance is supported by clinical studies in which topical delivery of muscarinic antagonists improved epidermal nerve fiber density in the skin of subjects with diabetic neuropathy [13,15].

Given the broadly similar clinical profiles, and possibly pathogenic mechanisms, of peripheral neuropathy in patients with diabetes and CIPN, the present studies were undertaken to assess the therapeutic potential of the M_1_R selective antagonist pirenzepine as a treatment for CIPN and to provide preclinical guidance for the design of anticipated clinical trials. This therapeutic approach to treatment of CIPN is supported by studies which demonstrated that the M_1_R/M_3_R antagonist benztropine prevented multiple functional and structural indices of neuropathy in mice when given concurrently with oxaliplatin [16], and that the specific M_1_R antagonist muscarinic toxin 7 (MT-7) reversed sensory nerve loss in the cornea of oxaliplatin-exposed mice [10]. As neuropathic pain is the primary feature of CIPN that drives patients to moderate, or withdraw from, their optimal chemotherapeutic regimen, we adopted allodynia to light touch of von Frey filaments as the primary readout for both onset of neuropathy and efficacy of pirenzepine treatment.

## 2. Materials and Methods

### 2.1. Animal Models

Adult (20–30 g) female (*n* = 264) or male (*n* = 50) Swiss Webster mice (Charles River, Hollister, CA, USA) were used under protocols approved by the local Institutional Animal Care and Use Committee. Of the 314 mice initiated across 8 separate studies, there were 8 within-study deaths, of which 3 were of undetermined cause in female mice during acclimation following arrival at the vivarium and 5 were male mice following initial injection of bortezomib. Mice were maintained 3–5/cage in an AAALAC-approved vivarium under a 12 h light:dark cycle with free access to food (5001 diet, Purina LabDiet, Gray Summit, MO, USA) and water. Throughout the studies, mice were observed daily in their home cage for signs of distress (posture, fur, and mobility) or physical injury and weighed weekly. No deviation from normal behavior or adverse events were recorded. Oxaliplatin-induced peripheral neuropathy was induced by intraperitoneal (i.p.) injection of oxaliplatin dissolved in a 5.0% glucose, 0.9% saline solution at a dose of 5 mg/kg on alternate days to a cumulative dose of 20 mg/kg, as previously used to produce neuropathy characterized by allodynia to light touch and reduced corneal nerve density [10]. Paclitaxel-induced peripheral neuropathy was induced by i.p. injection of 1 mg/kg or 4 mg/kg paclitaxel dissolved in a vehicle comprising Kolliphor EL:ethanol:saline (1:1:2 volume ratio) on alternate days over 9 days to a cumulative dose of 5 or 20 mg/kg, as previously used to produce neuropathy characterized by touch-evoked allodynia and heat hyperalgesia [7]. Bortezomib-induced peripheral neuropathy was induced by i.p. injection of bortezomib dissolved in 0.9% saline at a dose of 0.5 or 1.0 mg/kg on alternate days to a cumulative dose of 3 mg/kg as reported to produce heat hyperalgesia [17]. In proof-of-concept studies, mice received daily (5/week unless stated) systemic treatment with pirenzepine dihydrochloride (Sigma: 0.1–10.0 mg/kg s.c.) dissolved in sterile 0.9% saline or vehicle alone. This dose range was based on prior efficacy studies in rodents with diabetic neuropathy [7]. Treatment began either 3 days prior to the chemotherapeutic regimen (prevention) or after indices of neuropathy were detected (intervention). Topical delivery of pirenzepine was also performed to explore future clinical use, as topical pirenzepine has been reported to reverse indices of neuropathy in humans with diabetic neuropathy [14]. For topical delivery, pirenzepine was mixed into a volume of hydrogel (Intrasite, Smith & Nephew, Hull, UK) and 50 µL of 1–10% preparation applied to the plantar hind paw, which was subsequently inserted into a plastic glove-like cover to prevent animals ingesting or removing the application. After 30 min, the protection was removed and the hydrogel wiped away. Treatment was performed 5 days per week, with the dose range based on prior efficacy studies in diabetic rodents [14].

To evaluate whether pirenzepine impacted the cytotoxic efficacy of chemotherapeutic agents, 8-week-old female nu/nu mice (Charles River, Hollister, CA, USA) were injected with human ovarian carcinoma cells (OVCAR-8: 10^7^ cells i.p. [18]). After one week, mice were treated with 6 cycles of a cisplatin (2 mg/kg i.p.) and paclitaxel (10 mg/kg i.p.) cocktail, which was given twice weekly for three weeks. Mice were also treated with pirenzepine (10 mg/kg/day s.c.) or saline vehicle. Mice were harvested at 4 weeks and peritoneal tumors excised and weighed in aggregate.

### 2.2. Cell Culture

Triple-negative breast cancer cells (MDA-MD-468 and MDA-MB-231) and the ovarian cancer cell line OVCAR-3 were used as these indications are commonly treated with agents that cause CIPN such as taxanes and platins. As a contrast, the neuroblastoma line Kelly was also evaluated. Cells were exposed to vehicle (control) or a paclitaxel for 72 h in the presence or absence of 1μM pirenzepine. Surviving cells were quantified by staining with crystal violet and viewing by fluorescence microscopy at 600 nm before calculation of the LD_50_.

### 2.3. Evoked Responses

To measure paw sensitivity to light touch, mice were transferred to a testing cage with a wire mesh bottom and allowed to acclimate for 5–15 min. Von Frey filaments (Stoelting, Wood Dale, IL, USA) were applied in sequence to the plantar surface of the hind paw with a pressure that caused the filament to buckle [19]. Lifting of the paw was recorded as a positive response and the next lightest filament chosen for the next measurement. Absence of a response after 5 s prompted use of the next filament of increasing weight. This paradigm was continued until four measurements had been made after an initial change in the behavior or until five consecutive negative or four positive scores had occurred. The resulting sequence of positive and negative scores was used to interpolate the 50% paw response threshold [20].

To measure heat pain perception, mice were placed in an observation chamber on the surface (floor temperature 30 °C) of a modified Hargreaves Apparatus (UARD, San Diego, CA, USA) and allowed to acclimate for 5–15 min. A heat source (surface heating rate of ~1 °C/s) was maneuvered underneath the hind paw and activated. The heat source and associated timer shut off automatically when the paw was withdrawn or at the 20 s maximal cut off time. The procedure was repeated 3 times on the same paw at 5 min intervals and the median used to represent the paw thermal response latency. In some studies, a time vs. surface temperature standard curve was constructed immediately before testing to allow response temperature to be interpolated from the response time [19].

### 2.4. Nerve Conduction Velocity

Mice were anesthetized with isoflurane and needle stimulating electrodes placed at the sciatic notch and Achilles tendon of the hind limb with recording electrodes placed in the interosseus muscles of the ipsilateral foot. Nerve temperature was maintained at 37 °C and the nerve stimulated by single square-wave pulses applied first to the notch and then the Achilles tendon. Peak–peak latency of the resulting M waves was used to derive MNCV using the distance between stimulating electrodes [19].

### 2.5. Data Handling and Analysis

Following a 1-week acclimation period, mice were randomly (no inclusion/exclusion criteria applied other than still alive) assigned to experimental groups by blind distribution. Mice, home cages and treatments were coded to blind the persons performing tests to the study group assigned to each animal. Animal, cage and treatment codes were held by 1 person not directly involved in treatment or data collection. Cages were drawn at random for testing. Group sizes were determined by prior experience with the assays to be used and the magnitude of CIPN-induced allodynia as established in pilot studies. Comparisons between 2 groups were performed by unpaired *t* test, whereas data sets containing 3 or more groups were analyzed using Prism 11.0.2 software (Graphpad, San Diego, CA, USA) by one-way or repeated-measures ANOVA, as appropriate for the experimental design. Between-group differences were identified by the Holm–Sidak *post hoc* test. Group size and the specific statistical test used are described in figure legends.

## 3. Results

### 3.1. Oxaliplatin-Induced Neuropathy

Paw withdrawal from von Frey filaments was measured as the primary index of neuropathic pain in adult female Swiss Webster mice before and after receiving systemic injections of vehicle (0.9% saline) or oxaliplatin (5 mg/kg on alternate days to a cumulative dose of 20 mg/kg). The study was designed *a priori* to investigate change from pre-treatment values for all groups. Oxaliplatin induced acute tactile allodynia, as measured immediately at the end of the treatment regimen (*p* < 0.001 vs. pre-treatment values), that partially resolved over time, although values remained significantly (*p* < 0.05) lower than pre-treatment values by study end at week 15 (Figure 1A). Mice that received daily treatment with pirenzepine (1 mg/kg/day s.c.) beginning 3 days prior to onset of the oxaliplatin regimen and continuing throughout the study also showed significant (*p* < 0.001 vs. pre-treatment values) allodynia. Allodynia resolved so that values were not significantly different from pre-treatment values at weeks 9 and 15 of the study.

To determine whether resolution of allodynia in oxaliplatin-exposed pirenzepine-treated mice reflected an acute analgesic effect of pirenzepine, previously vehicle-treated control and oxaliplatin-exposed mice received a single treatment with pirenzepine (1 mg/kg s.c.). There was no effect on paw withdrawal latency for up to 6 h post-treatment in either control or oxaliplatin-exposed mice when compared to pre-treatment values (Figure 1B).

A second study was performed in oxaliplatin-exposed adult female Swiss Webster mice to assess (i) efficacy of pirenzepine when delivered topically to the paw, (ii) efficacy of pirenzepine treatment when initiated only after onset of oxaliplatin-induced allodynia and (iii) persistence of efficacy after withdrawal of pirenzepine. The study was designed *a priori* to measure differences vs. the oxaliplatin + vehicle group at multiple time points. Oxaliplatin (5 mg/kg on alternate days to a cumulative dose of 20 mg/kg) induced significant tactile allodynia at the end of the treatment regimen (Figure 2A: week 0, control + vehicle vs. oxaliplatin + vehicle: *p* < 0.001), which persisted for at least 12 weeks (*p* < 0.05 at week 12) before resolving. Topical pirenzepine treatment (50 µL of 4% in hydrogel) gradually alleviated allodynia in oxaliplatin-exposed mice so that after 4 weeks of topical daily treatment, values were significantly (*p* < 0.001) higher than vehicle-treated, oxaliplatin-exposed mice. At this point, pirenzepine treatment was stopped. Nevertheless, values remained significantly elevated compared to vehicle-treated, oxaliplatin-exposed mice for the following 5 weeks (*p* < 0.01 at week 9), after which values converged with the vehicle-treated oxaliplatin-exposed mice.

Measurements of sensory neuropathy were extended to include paw withdrawal latency to escalating heat, which was measured at week 7 following cessation of the oxaliplatin regimen and thus 3 weeks after the last topical pirenzepine treatment (Figure 2B). Vehicle-treated, oxaliplatin-exposed mice showed significant heat hyperalgesia (*p* < 0.01) compared to vehicle-treated control mice, and this was completely prevented by daily topical pirenzepine treatment (*p* < 0.01 vs. vehicle-treated, oxaliplatin-exposed mice).

### 3.2. Paclitaxel-Induced Neuropathy

Paw withdrawal threshold was measured as the primary index of neuropathic pain in adult female Swiss Webster mice exposed to a regime of injections of vehicle (0.9% saline) or paclitaxel (1 mg/kg prepared in 1:1:2 Kolliphor EL:ethanol:saline on alternate days to a cumulative dose of 5 mg/kg over 9 days). After paclitaxel exposure was concluded and the presence of allodynia to light touch established, mice began daily treatment with pirenzepine (0.1, 1.0 or 10.0 mg/kg/day s.c.) or vehicle (0.9% saline) for 8 weeks. The study was designed *a priori* to measure differences between the vehicle-treated, paclitaxel-exposed mice and all other groups at multiple time points. Following paclitaxel exposure, paw withdrawal threshold from von Frey filaments was significantly (*p* < 0.001: Figure 3A) different from control mice. This allodynia remained significant throughout the 8-week study period (*p* < 0.001, all time points measured). Paclitaxel-exposed mice treated with 0.1 mg/kg/day pirenzepine also remained allodynic throughout the study and were not significantly different from the vehicle-treated, paclitaxel-exposed mice at any time. In contrast, mice treated with 1.0 or 10.0 mg/kg/day pirenzepine showed alleviation of allodynia as early as 2 weeks after onset of treatment (*p* < 0.01 for 1.0 mg/kg/day group vs. vehicle-treated group), which persisted throughout the study. It is notable that whereas the 1.0 mg/kg/day treated group showed statistically significant divergence from the vehicle-treated group at all time points measured, the 10.0 mg/kg/day was limited to statistical significance (*p* < 0.001) only after 8 weeks of treatment. At study end, other indices of peripheral neuropathy were also assessed. Vehicle-treated, paclitaxel-exposed mice showed paw hyperalgesia to heat (*p* < 0.05 vs. control + vehicle-treated mice), which was dose-dependently alleviated by pirenzepine treatment (Figure 3B), with the 10 mg/kg/day group showing statistical significance (*p* < 0.05 vs. vehicle-treated, paclitaxel-exposed mice). Vehicle-treated, paclitaxel-exposed mice also showed reduced MNCV slowing (*p* < 0.05 vs. control + vehicle) that was increased by all doses of pirenzepine (Figure 3C), while rotarod performance was not different in any group (Figure 3D).

In a separate series of experiments designed to guide future clinical studies, we explored optimal delivery profiles for efficacy against allodynia in paclitaxel-exposed female mice treated topically with pirenzepine. Mice received daily paw application of pirenzepine (50 µL of 10.0% in hydrogel, 30 min/day, 5 days/week) starting either 3 days prior to (prevention) or after (intervention) completion of the paclitaxel regimen (4 mg/kg prepared as above and given on alternate days to a cumulative dose of 20 mg/kg over 9 days). The study was designed to identify within-group change from values at completion of the paclitaxel regimen. Both groups of pirenzepine-treated mice, whether treated before or after the paclitaxel regimen, showed significant allodynia following completion of the paclitaxel regimen (*p* < 0.05 or *p* < 0.01 vs. pre-paclitaxel values), which resolved within 2 (prevention: *p* < 0.05) or 3 (intervention: *p* < 0.05) weeks after completion of the paclitaxel regimen (Figure 4A). In a second study, paclitaxel-exposed female mice (cumulative dose 20 mg/kg as above) were treated topically to the paw with 1.0%, 3.0% or 10.0% pirenzepine daily, 5 days/week for 6 weeks, beginning two weeks after completion of the paclitaxel regimen. The study was designed to identify within-group change from values measured at completion of the paclitaxel regimen. Control mice (no paclitaxel, no pirenzepine) and paclitaxel-exposed mice treated with 1.0% pirenzepine did not significantly change paw withdrawal thresholds across the study (Figure 4B). While paw withdrawal threshold of paclitaxel-exposed mice treated with either 3% or 10% pirenzepine did not change between values pre-treatment and those measured after 2 weeks of treatment, both groups showed a significant (*p* < 0.05 and *p* < 0.01, respectively, for the 3% and 10% groups) increase from pre-treatment values after 6 weeks of treatment to approximate those of the control mice. In a third study, adult male mice were introduced to the protocol and the efficacy of different pirenzepine delivery frequencies assessed. Paclitaxel-exposed mice (cumulative dose 20 mg/kg as above) were treated topically to the paw with 10% pirenzepine in hydrogel either 1, 3 or 5 days/week for 3 weeks, starting immediately after completion of the paclitaxel regimen and confirmation of allodynia. The study was designed to identify within-group change from values at completion of the paclitaxel regimen. The paw withdrawal threshold of control mice (no paclitaxel, no pirenzepine) remained stable across the study (Figure 4C), while all paclitaxel-exposed mice showed significant allodynia at the end of the treatment regimen (*p* < 0.05 or less vs. pre-paclitaxel values), which was comparable to that of female paclitaxel-exposed mice (Figure 4A,B). Allodynia persisted for 3 weeks in paclitaxel-exposed mice that did not receive any pirenzepine treatment, whereas topical treatment with pirenzepine at a frequency of 1, 3 or 5 days/week significantly alleviated allodynia (all *p* < 0.05 vs. within-group pre-treatment values).

### 3.3. Bortezomib-Induced Neuropathy

Adult female Swiss Webster mice were exposed to bortezomib (1 mg/kg in sterile 0.9% saline on alternate days to a cumulative dose of 3 mg/kg over 3 days) and treated with vehicle or pirenzepine (10 mg/kg/day s.c.) for 3 days prior to onset of bortezomib treatment. Five mice died of unconfirmed cause on the first day following bortezomib treatment and none thereafter. Paw withdrawal threshold from von Frey filaments was measured as the primary index of neuropathic pain prior to onset of pirenzepine treatment (t = −2) and 2 and 4 weeks later. This study was designed *a priori* to investigate change from pre-treatment values for all groups. The paw withdrawal threshold of control mice (no pirenzepine, no bortezomib) did not significantly vary over time, whereas mice exposed to bortezomib, either with or without concurrent pirenzepine treatment, showed significantly reduced response thresholds at week 0 (both *p* < 0.01 vs. pre-bortezomib values) that resolved by week 2 (Figure 5). Pirenzepine-naive bortezomib-exposed mice then received a second round of bortezomib (0.5 mg/kg on alternate days to a cumulative dose of 2 mg/kg over 7 days), and allodynia was confirmed before starting treatment with either pirenzepine (10 mg/kg/day s.c.) or vehicle (t = 6). Pirenzepine treatment was without effect on paw withdrawal threshold of bortezomib-exposed mice after either 2 (t = 8) or 4 (t = 10) weeks of treatment.

### 3.4. Cytotoxicity

Exposure of MDA-MD-468, MDA-MB-23 or OVCAR-3 cell lines to a paclitaxel dose range for 72 h resulted in dose-dependent cell death and an LD_50_ that was not impacted by concurrent exposure to 1 µm pirenzepine (Figure 6A,B). In contrast, the LD_50_ for paclitaxel-induced killing of KELLY neuroblastoma cells was significantly (*p* < 0.01) increased in the presence of pirenzepine (Figure 6B). At autopsy, the body cavity of mice injected with OVCAR-8 cells included multiple white nodules shaped like rice grains throughout the body cavity and tumors embedded in adipose tissue. The tumor burden (total weight of nodules and tumors extracted) was markedly reduced in mice that received the cisplatin/paclitaxel cocktail and limited to occasional sporadic nodules. The efficacy of the chemotherapeutic cocktail in reducing tumor burden was not altered by co-treatment with pirenzepine (Figure 6C).

## 4. Discussion

Peripheral neuropathies, while originating from distinct insults, are increasingly recognized as sharing common pathogenic features [6], of which the physical and functional disruption of mitochondria appears central to progression of pain, nerve damage and degeneration [5,21]. For example, mitochondrial dysfunction is widely reported in animal models of diabetic neuropathy [22], and treating diabetic rodents with agents that activate mitochondria, such as M_1_R antagonists and other AMPK activators, produces multiple beneficial effects [7,23]. M_1_R inhibition, acting in its canonical role of stabilizing local PIP_2_ turnover, activates neuronal TRPM3 channels, resulting in a slow rise in cellular calcium that triggers a CAM-kinase cascade leading to activation of AMPK [8,9], a master regulator of mitochondrial number, quality and function [24]. Increased energy production is accompanied by activation of a parallel pathway arising from biased agonism at the M_1_R that promotes β-arrestin binding and cascades to drive regenerative growth within an environment of increased local energy production [11,25]. Given the ability of muscarinic receptor antagonists to prevent and reverse indices of peripheral neuropathy in rodent models of diabetes [7,14] and recent proof-of-concept studies in human subjects with diabetic neuropathy [13,15], it seemed reasonable to extend this potential therapeutic approach into rodent models of CIPN, which also show damaged mitochondria and disruption of the cytoskeletal organization necessary for growth and maintenance of the distal axon [4,26].

Peripheral neuropathy markedly impedes quality of life for those receiving chemotherapy, while neuropathic pain may also precipitate dose reduction or truncation of the optimal chemotherapy regimen. We therefore focused on allodynia to the light touch of von Frey filaments as a large fiber-mediated, clinically relevant primary readout of efficacy, as it is a common feature of multiple rodent models of CIPN [27] and widely used to assess efficacy of potential therapeutics designed to prevent or alleviate pain in CIPN [28]. Allodynia developed within days of exposure to oxaliplatin, paclitaxel or bortezomib and persisted for weeks to months after the chemotherapy regimen ceased. Onset of allodynia was not prevented by pre-treatment with pirenzepine in any model of CIPN tested. This is consistent with its failure as an acute-onset analgesic in mice with oxaliplatin CIPN (this study) or in rats with diabetic neuropathy in which it had neither analgesic nor sedative properties [7]. The onset of pain in models of CIPN may be analogous to the acute pain syndrome seen in patients undergoing treatment with oxaliplatin or paclitaxel [29,30], with proposed neurotoxic mechanisms that include onset of aberrant ion channel expression and nociceptor excitability [28]. In contrast, pirenzepine treatment, whether begun before or after the chemotherapeutic regimen, accelerated the inherent recovery from allodynia seen over the weeks following exposure to oxaliplatin or paclitaxel. While pirenzepine may have diverse effects when delivered systemically due to the widespread distribution of the M_1_R, including parasympathetic cholinergic systems, the lack of impact of pirenzepine on rotarod performance after chronic treatment (Figure 3) and prior reports that there is no acute effect on rotarod performance following a single delivery [7] suggest that the increase in response threshold to von Frey filaments is not the result of sedation or other impediments to motor response capability. The delayed onset of pirenzepine’s effect on allodynia is consistent with the concept of the evolution of pain in which initiating mechanisms transition into those that sustain neuropathic pain well beyond the exposure to chemotherapeutics. Mechanisms proposed to contribute to neuropathic pain in CIPN include neuroinflammation, mitochondrial dysfunction and damage, cytoskeletal disruption and DNA damage [2]. Whether pirenzepine reverses allodynia by preventing this mechanistic transition or is effective only on mechanisms contributing to later phases of pain maintenance requires further studies. However, given the capacity of M_1_R antagonists to both enhance mitochondrial function and promote axonal growth, it is plausible that alleviation of allodynia in the oxaliplatin and paclitaxel models represents accelerated neuronal repair and recovery from injury, consistent with restored sensory nerve density in the cornea of oxaliplatin-exposed mice by the selective M_1_R antagonist MT-7 [10].

While allodynia to light touch is common to many animal models of CIPN, the overall neuropathy phenotype can vary with the chemotherapeutic agent used, the severity of the dosing regimen employed and the mouse background strain [27]. Both our oxaliplatin and paclitaxel models displayed hyperalgesia to escalating heat that illustrates an evoked pain-like state mediated by small sensory fibers. Heat hyperalgesia, like touch-evoked allodynia, was absent in both oxaliplatin- and paclitaxel-exposed mice treated with pirenzepine, though the lack of measurements across time prevents interpretation of efficacy as either prevention or reversal of hyperalgesia. In the case of oxaliplatin-induced heat hyperalgesia, the effect of pirenzepine was measured after 4 weeks of daily topical treatment followed by 3 weeks of withdrawal from treatment. As with allodynia in these animals, the impact of pirenzepine does not require immediate drug presence and persists for a substantial time. Both paclitaxel-induced heat hyperalgesia and tactile allodynia also showed dose-dependent responses to pirenzepine. In contrast, the conduction slowing of large, myelinated motor fibers seen in paclitaxel-exposed mice did not closely match other indices of neuropathy in the same animals, as pirenzepine was effective against this disorder at the lowest dose given (0.1 mg/kg/day), whereas this dose was ineffective against touch-evoked allodynia and heat hyperalgesia in the same animals. Whether this indicates greater potency in motor compared to sensory fibers or heterogenicity of mechanisms driving increased sensation versus slowed conduction remains to be explored. It will also be of value to explore structural indices of neuronal degeneration and regeneration such as small sensory nerve fiber density in the skin and cornea and morphometric parameters of large sensory and motor fibers in nerve trunks.

It is notable that pirenzepine treatment, in either prevention or intervention paradigms, was without effect on allodynia in bortezomib-exposed mice. Oxaliplatin, paclitaxel and bortezomib initiate cytotoxicity by different primary insults but share several downstream consequences that contribute to neurotoxicity and CIPN, including mitochondrial damage and dysfunction [4]. Our premise that muscarinic antagonism would be effective in all peripheral neuropathies in which mitochondrial dysfunction contributes is therefore not supported. This could reflect technical issues in our bortezomib-induced CIPN model, which used a notably high-dose and short-duration exposure paradigm [17] compared to other studies [31] and could produce excess physical injury to mitochondria that would not be responsive to AMPK-mediated activation provoked by pirenzepine. Diverse agents known to enhance mitochondrial activity attenuated allodynia in mice exposed to a less aggressive bortezomib regimen of 0.2 mg/kg on alternate days to a cumulative dose of 1 mg/kg [32]. Evaluation of mitochondrial structure and function in our bortezomib model is warranted. We may also have used a sub-optimal systemic pirenzepine dose (see Figure 3A) so that dose range studies are warranted. It is also plausible that the efficacy of pirenzepine in oxaliplatin and paclitaxel models is not related to mitochondrial activation and that whatever mechanism is involved in accelerated recovery from CIPN does not pertain to bortezomib CIPN. Additional mechanistic studies are clearly required.

These preclinical studies were performed to guide the design of potential future clinical studies intended to reduce pain and allow continued optimal chemotherapeutic dosing in subjects with CIPN. Efficacy of pirenzepine against established allodynia allows an interventional design in subjects with established neuropathic pain. This removes the need for the large study cohorts necessary in pain prevention studies, where not all subjects would naturally progress to painful neuropathy, simplifying study design and analysis while reducing study cost. Consideration of whether to target those with recent onset of pain or include subjects with established pain may also be warranted. The efficacy of topical delivery is also beneficial as this route reduces the risk of systemic side effects that are inherent to muscarinic antagonists, while anti-muscarinic impact on the CNS is unlikely given the poor blood–brain–barrier penetration of pirenzepine [33]. There is precedence for an acceptable safety profile, as topical delivery of daily 4% pirenzepine to subjects with diabetic neuropathy for many months produced minimal adverse effects [15]. Another potentially helpful feature emerging from these studies is the duration of efficacy. While pirenzepine is not an acute analgesic and alleviation of allodynia took weeks of daily treatment to emerge, efficacy also persisted for weeks after withdrawal of treatment until converging with the natural recovery that developed in vehicle-treated CIPN animals. This suggests that pirenzepine treatment initiates a long-acting change in neuronal phenotype, possibly by accelerating endogenous recovery mechanisms [11,12]. It also offers the potential for tapering treatment frequency and/or dose once pain relief is achieved, particularly as interventional efficacy was also seen when treatment was reduced to once weekly. While it can be difficult to maintain subject compliance when a pain-relieving agent lacks immediate effect and requires extended treatment, the potential for long-acting efficacy and treatment tapering/cessation after the end of chemotherapy exposure cycles can be an attractive proposition. Continuing pirenzepine treatment through subsequent cycles of chemotherapy is not likely to impair cytotoxic efficacy, as pirenzepine did not impede paclitaxel-induced killing of breast or ovarian cell cancer lines in vitro or reduction in tumor burden by a chemotherapeutic cocktail after seeding mice with an ovarian cancer cell line. The cell lines used represent a variety of tumor types and levels of M_1_R expression, with the triple-negative breast cancer cells expressing moderate (MDA-MD-468) or low (MDA-MB-231) levels of M_1_R, while the ovarian cancer cell line OVCAR3 does not express M_1_R. The one cell line in which paclitaxel LD_50_ was increased was a neuroblastoma, which expresses moderate-to-high levels of M_1_R. The clinical impact of this is unclear as neuroblastomas are resistant to taxanes (note the high LD_50_ compared to other cells studied), and taxanes are accordingly not used in treatment of neuroblastoma. It is plausible that pirenzepine was activating neuronal survival and recovery mechanisms in this cell line, as previously reported for adult rodent sensory neurons exposed to paclitaxel or oxaliplatin [7].

Limitations of the studies reported above include the unresolved pertinence of mouse models of CIPN to the human condition, the lack of concurrent cancers in the CIPN models, the incomplete replication of all studies in both sexes and reliance on evoked responses to touch or heat that, while pertinent to human CIPN, may not represent other aspects of the condition such as spontaneous pain. Future studies could mitigate these concerns by expanding to a full exploration in both sexes and other non-human species where the animals are also tumor-bearing and by introducing assays considered more representative of spontaneous pain such as operant and conditioned place preference/avoidance assays [34].

## 5. Conclusions

To date, therapeutic approaches that have advanced to clinical use for preventing or reversing neuropathic pain have been largely limited to short-acting analgesics, which can be of variable and unpredictable efficacy, induce addictive or other harmful side effects, do not address the underlying neurodegeneration and are thus not disease-modifying. Our studies identify pirenzepine as a potential therapeutic against indices of both neuropathic pain and neuropathy induced by oxaliplatin or paclitaxel. Pirenzepine does not prevent onset of pain or act as an acute analgesic but gradually alleviates allodynia over weeks of treatment in parallel with preventing or reversing nerve conduction slowing, a measure of degenerative neuropathy. These preclinical data position pirenzepine for future clinical studies and suggest an optimal treatment paradigm involving topical delivery to subjects who have already developed painful CIPN with the potential for tapering the treatment dose/frequency once pain relief is established.

## Figures and Tables

**Figure 1:**
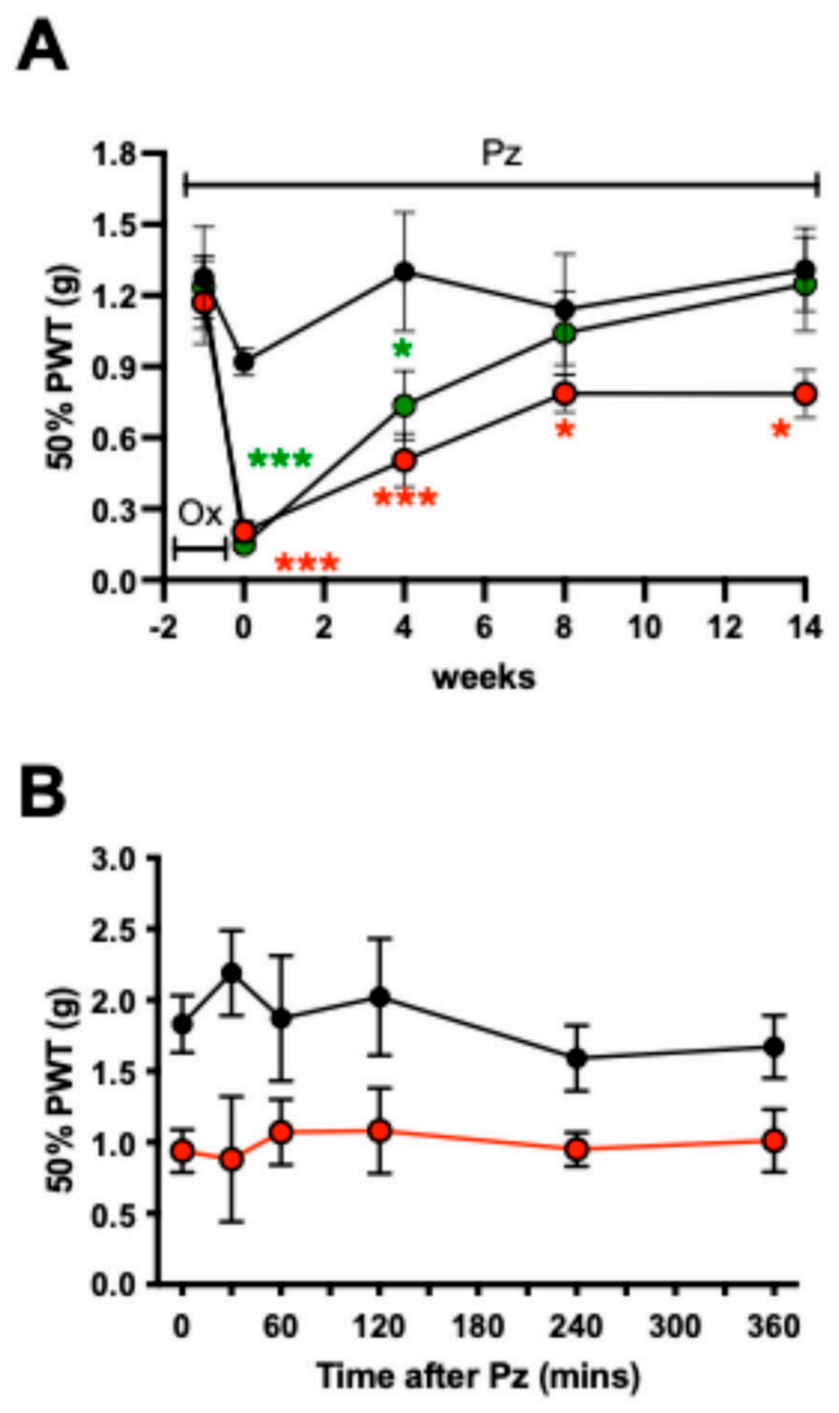
Pirenzepine accelerates recovery in oxaliplatin CIPN. (**A**) 50% paw withdrawal threshold (PWT) in control (*n* = 10, black symbols), oxaliplatin-exposed (*n* = 10, red symbols) and oxaliplatin-exposed + treatment with pirenzepine (Pz: 1 mg/kg/day s.c.: *n* = 10, green symbols) mice starting 3 days before onset of oxaliplatin treatment. (**B**) 50% paw withdrawal threshold (PWT) in control (*n* = 8, black lines and symbols) and oxaliplatin-exposed (*n* = 9, red lines and symbols) mice before (t = 0) and following a single injection of pirenzepine (PZ: 1 mg/kg s.c.). Data are group mean ± SEM. Statistical analysis was by repeated-measures ANOVA with the Holm–Sidak *post hoc* test to compare within-group values against t = 0 h. * = *p* < 0.05, *** = *p* < 0.001 vs. t = 0 (pre-oxaliplatin).

**Figure 2:**
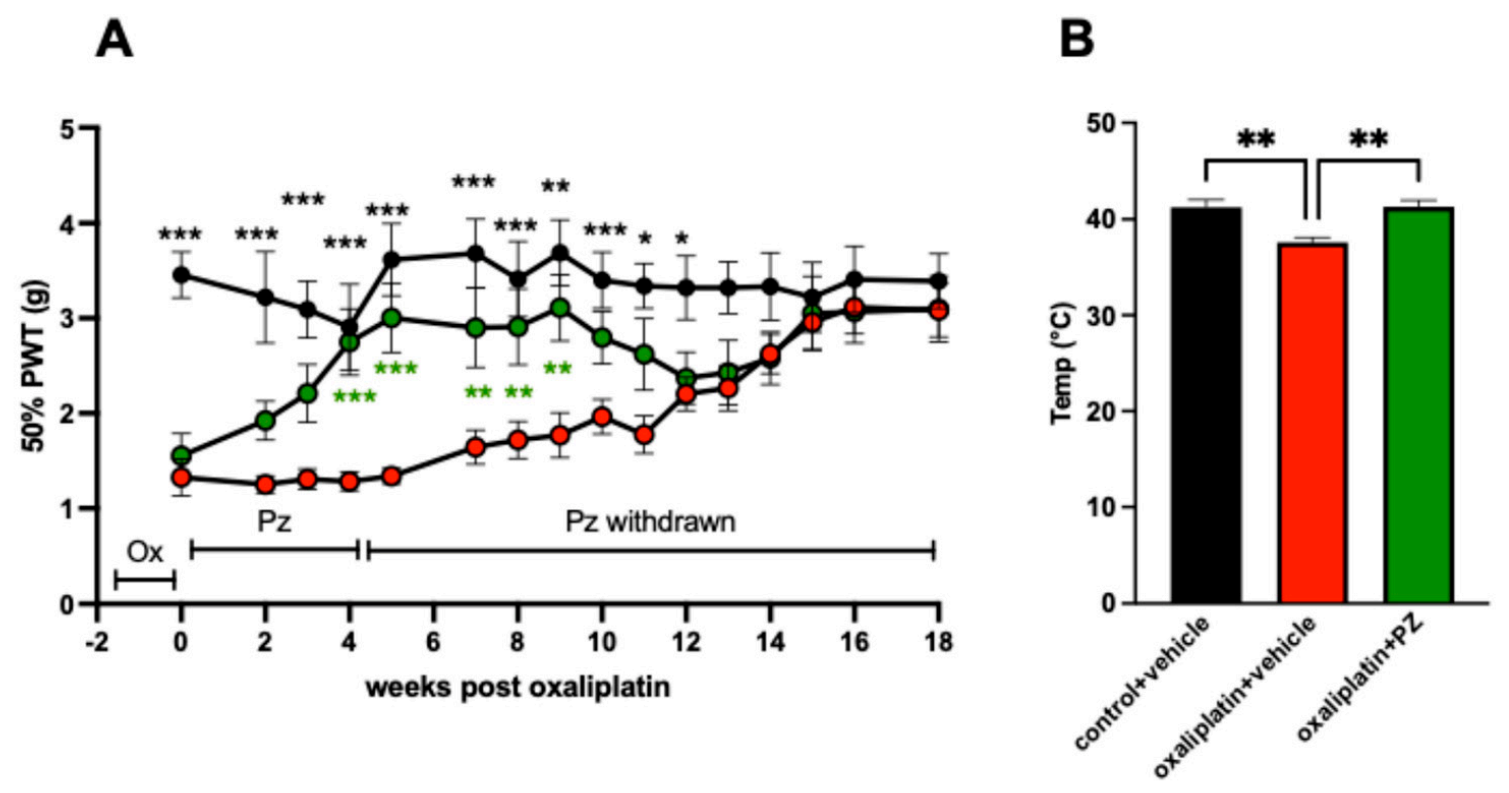
Topical pirenzepine reverses oxaliplatin CIPN and persists after cessation of treatment. (**A**) Paw withdrawal to von Frey filaments in control mice (*n* = 9, black symbols), oxaliplatin-exposed mice (*n* = 10, red symbols) and mice exposed to oxaliplatin then treated daily with topical pirenzepine (PZ: 4% gel to the hind paw) 30 min/day for 4 weeks before cessation of treatment (*n* = 10, green symbols). (**B**) Paw response temperature at study week 7 (bar colors represent the same groups as above). Data are mean ± SEM. Statistical analysis was by repeated-measures (panel (**A**)) or one-way (panel (**B**)) ANOVA with the Holm–Sidak *post hoc* test to compare against the oxaliplatin + vehicle group at each time point. * = *p* < 0.05, ** = *p* < 0.01, *** = *p* < 0.001.

**Figure 3:**
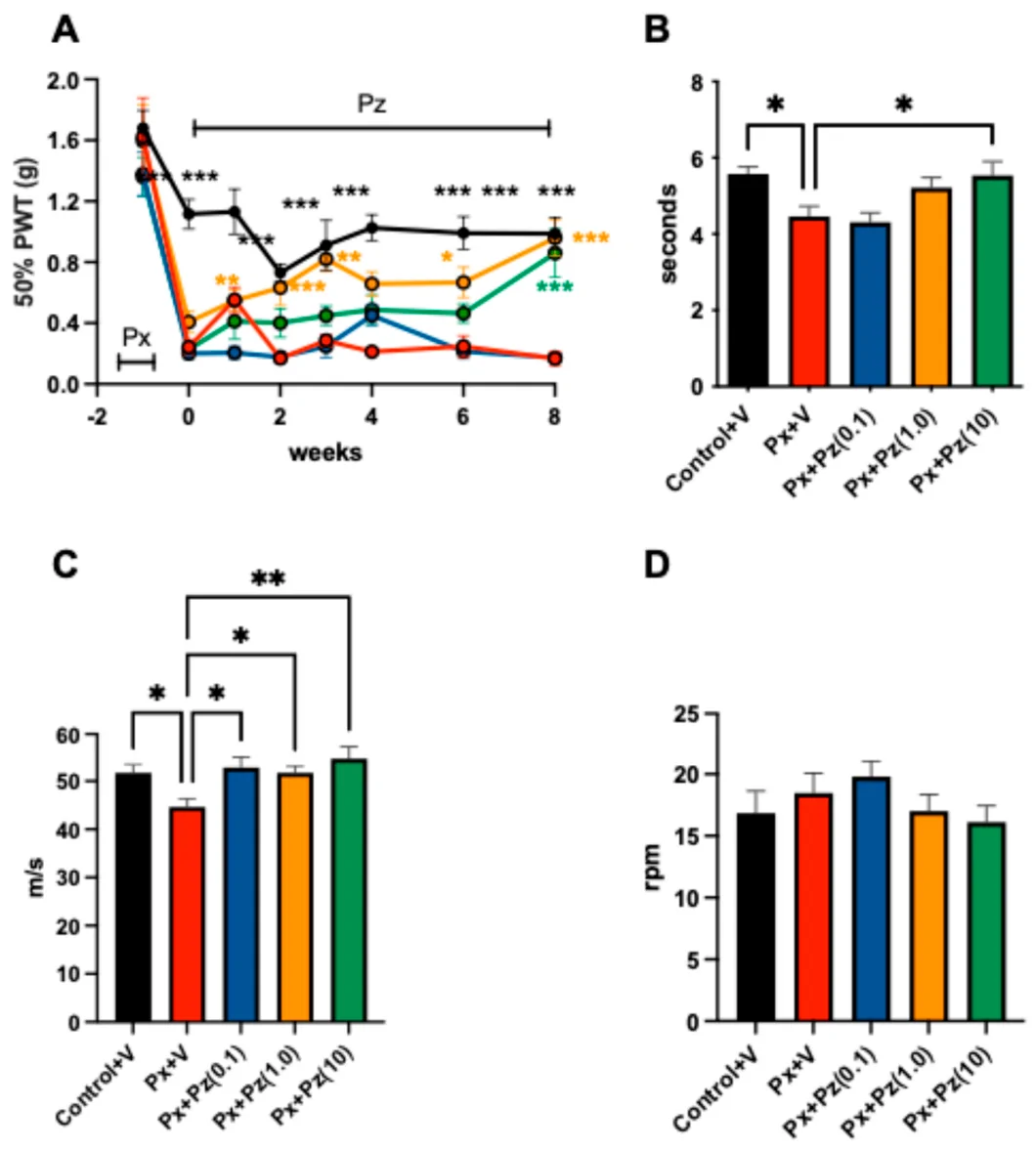
Pirenzepine reverses paclitaxel CIPN. Paw withdrawal threshold to von Frey filaments (panel (**A**)), paw withdrawal latency to heat (panel (**B**)), motor nerve conduction velocity (panel (**C**)) and rotarod performance (panel (**D**)) in control (*n* = 9, black line and bars) and paclitaxel (Px)-exposed mice followed by daily s.c. injection of vehicle (V: *n* = 10, red line and bars) or pirenzepine (Pz) at doses of 0.1 (*n* = 10, blue line and bars), 1.0 (*n* = 10, orange line and bars) or 10.0 (*n* = 10, green line and bars) mg/kg/day for 8 weeks. Data are group mean ± SEM. Statistical analysis was by repeated-measures (panel (**A**)) or one-way ANOVA (panels (**B**–**D**)) with the Holm–Sidak *post hoc* test to compare against the paclitaxel + vehicle group. * = *p* < 0.05, ** = *p* < 0.01, *** = *p* < 0.001 vs. paclitaxel + vehicle.

**Figure 4:**
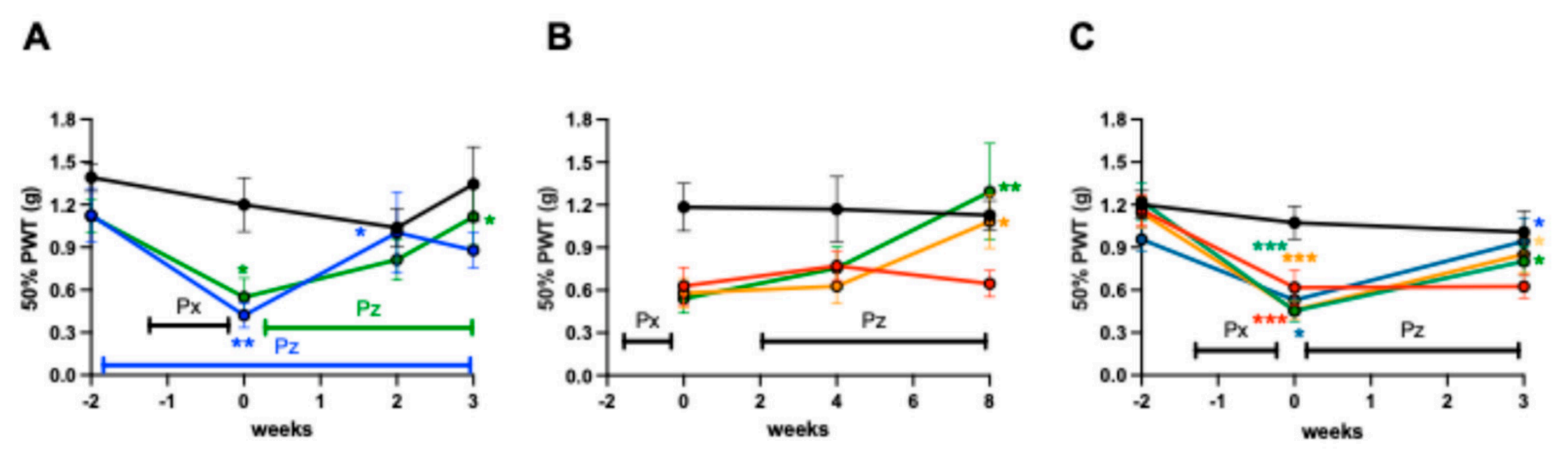
Topical pirenzepine reverses paclitaxel CIPN. Paw withdrawal threshold to von Frey filaments in control (*n* = 8–10, black lines) and paclitaxel (Px)-exposed mice followed by topical application of 50 µL vehicle (V) or pirenzepine (Pz) for 30 min/session. (**A**) Topical Pz (10% daily) was begun before (*n* = 8, blue lines) or after (*n* = 8, green lines) the Px exposure. (**B**) Topical Pz (daily) was begun 2 weeks after Px exposure at doses of 1% (*n* = 10, red lines), 3% (*n* = 10, orange lines) or 10% (*n* = 10, green lines). (**C**) Topical vehicle to control (*n* = 10, black lines, 5 days/week) or to paclitaxel-exposed (*n*=10, red lines) mice, and topical Pz (10%) to paclitaxel-exposed mice was begun 2 weeks after Px exposure with Pz given at frequencies of 1 (*n* = 10, green lines), 3 (*n* = 10, orange lines) or 5 (*n* = 10, blue lines) days/week. Data are group mean ± SEM. Statistical analysis was by repeated-measures ANOVA with the Holm–Sidak *post hoc* test to identify within-group change. * = *p* < 0.05, ** = *p* < 0.01, *** = *p* < 0.001.

**Figure 5:**
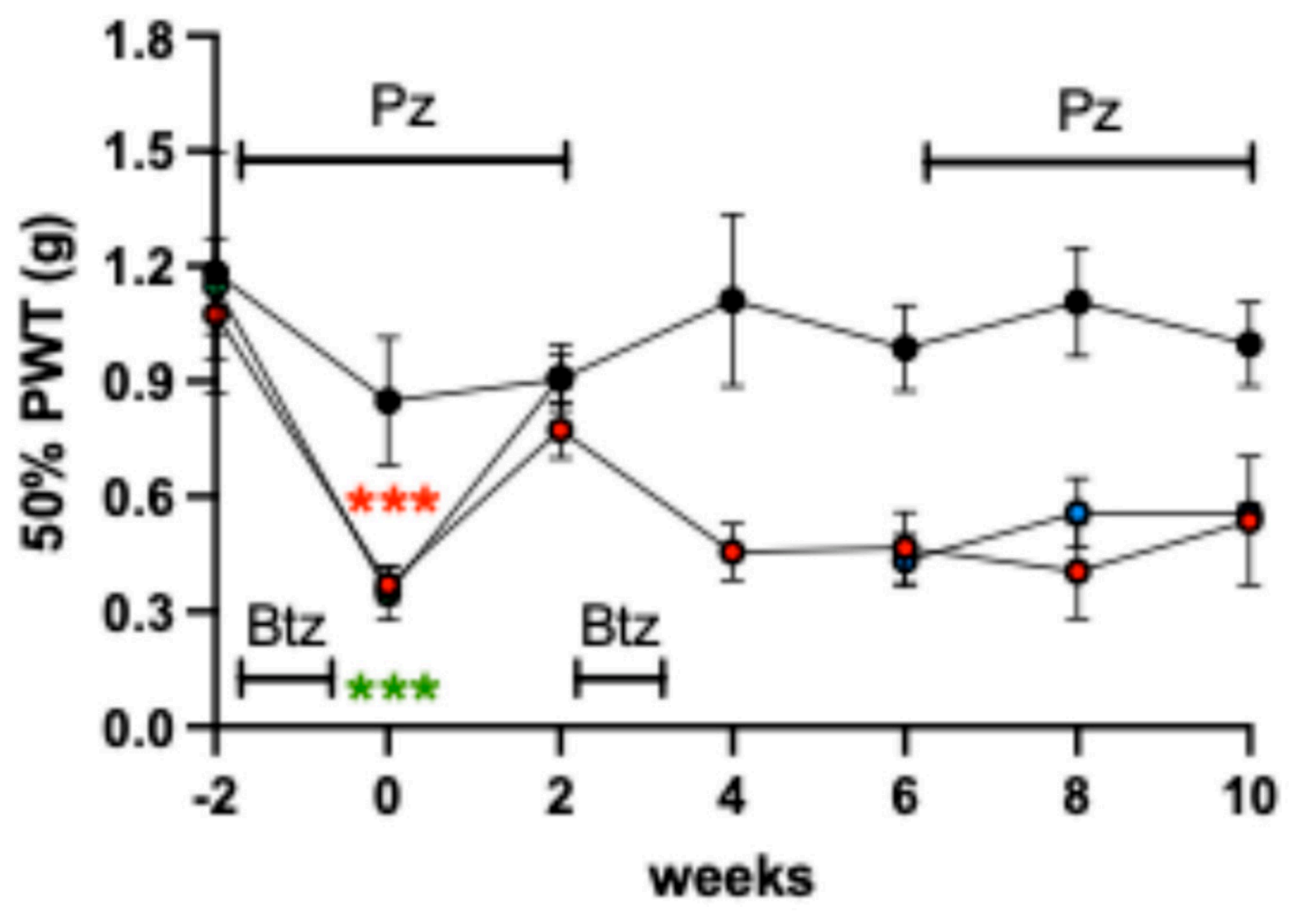
Pirenzepine does not prevent or reverse bortezomib CIPN. Paw withdrawal threshold to von Frey filaments in control (*n* = 10, black symbols) and bortezomib (Btz)-exposed mice followed by systemic delivery of vehicle (*n* = 9, red symbols) or pirenzepine (Pz 10 mg/kg/day s.c.) started before (*n* = 7, green symbols) or after (*n* = 9, blue symbols) Btz exposure. Data are group mean ± SEM. Statistical analysis was by repeated-measures ANOVA with the Holm–Sidak *post hoc* test to identify within-group change. *** = *p* < 0.001.

**Figure 6:**
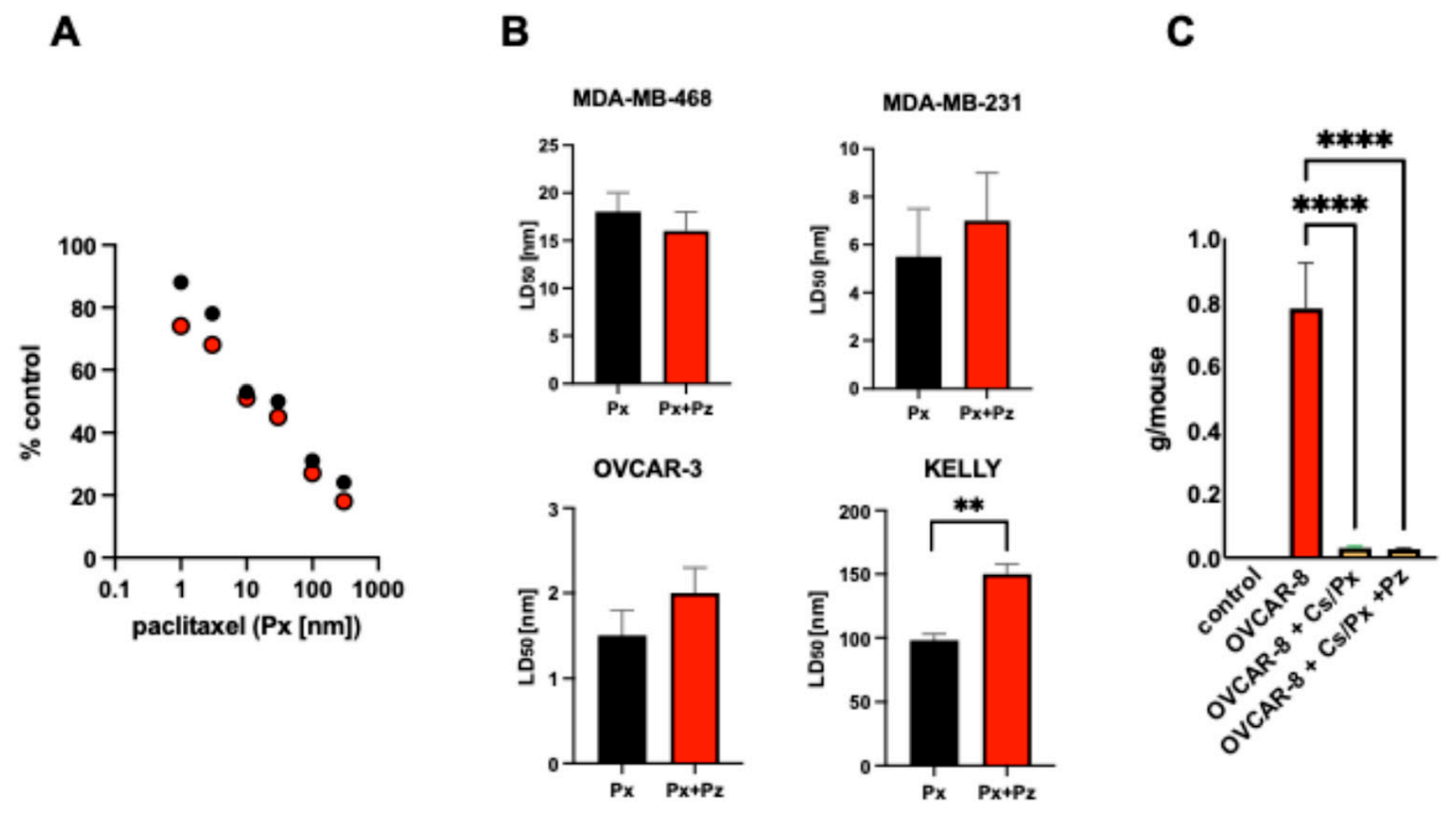
Pirenzepine did not impact the cytotoxicity of chemotherapeutic agents. (**A**) Survival (% control cell number) of MDA-MB-468 cells exposed to a paclitaxel (Px) dose range for 72 h (black symbols) ± 1 µM pirenzepine (Pz: red symbols). (**B**) LD_50_ for MDA-MB-468, MDA-MB-231, OVCAR-3 and KELLY cells exposed to the same dose range of paclitaxel (Px) ± 1µM Pz. (**C**) Tumor burden in mice injected with OVCAR-8 cells ± treatment with Px ± Pz. Data are mean + SEM of *n* = 3 replicates/data point (panels (**A**,**B**)) or mean + SEM of *n* = 10 mice/group (panel (**C**)) with statistical comparisons by unpaired *t* test (panel (**B**)) or one-way ANOVA with the Holm–Sidak *post hoc* test (panel (**C**)). ** = *p* < 0.01, **** = *p* < 0.0001.

## Data Availability

The data presented in this study are available on request from the corresponding author.

## Abbreviations

The following abbreviations are used in this manuscript:

CIPN	Chemotherapy-induced peripheral neuropathy
M_1_R	Muscarinic type 1 receptor
MNCV	Motor nerve conduction velocity
